# Titania-Catalyzed H_2_O_2_ Thermal Oxidation of Styrenes to Aldehydes

**DOI:** 10.3390/molecules24142520

**Published:** 2019-07-10

**Authors:** Satoru Ito, Yoshihiro Kon, Takuya Nakashima, Dachao Hong, Hideo Konno, Daisuke Ino, Kazuhiko Sato

**Affiliations:** 1 Institute for Energy and Material/Food Resources, Technology Innovation Division, Panasonic Corporation, 3-4 Hikaridai, Seika-cho, Soraku-gun, Kyoto 619-0237, Japan; 2 Interdisciplinary Research Center for Catalytic Chemistry, National Institute of Advanced Industrial Science and Technology (AIST), Central 5, 1-1-1 Higashi, Tsukuba, Ibaraki 305-8565, Japan

**Keywords:** titanium dioxide, hydrogen peroxide, styrenes, benzaldehydes, non-irradiated oxidation, heterogeneous catalyst

## Abstract

We investigated the selective oxidation of styrenes to benzaldehydes by using a non-irradiated TiO_2_–H_2_O_2_ catalytic system. The oxidation promotes multi-step reactions from styrenes, including the cleavage of a C=C double bond and the addition of an oxygen atom selectively and stepwise to provide the corresponding benzaldehydes in good yields (up to 72%). These reaction processes were spectroscopically shown by fluorescent measurements under the presence of competitive scavengers. The absence of the signal from OH radicals indicates the participation of other oxidants such as hydroperoxy radicals (•OOH) and superoxide radicals (•O_2_^−^) into the selective oxidation from styrene to benzaldehyde.

## 1. Introduction

The oxidation of olefins is widely known as a key reaction necessary for the production of various fine chemicals. In manufacturing, oxidation is used in versatile applications such as epoxidation, dihydroxylation and carboxylation [1]. Among them, the transformation from styrenes to benzaldehydes has attracted interest because of applicability of this process to the production of perfumes, pharmaceuticals and agrochemicals. This transformation is usually carried out through ozonolysis, which uses a toxic ozone as an oxidant and produces an explosive ozonide intermediate [2]. The oxidative cleavage of olefins by the OsO_4_–NaIO_4_ protocol generates hazardous heavy metal waste [3]. These classical methods have a severe impact on the environment. Therefore, a green alternative method for the oxidation of styrenes to benzaldehydes has been required [4,5].

Oxidation using hydrogen peroxide (H_2_O_2_) as an oxidant has a low environmental load due to high atom economy and only water as a byproduct. Heterogeneous catalysts, which are superior to homogeneous catalysts in both separability and reusability, have been developed for the activation of H_2_O_2_. Various heterogeneous catalysts for the H_2_O_2_ oxidation of styrenes to benzaldehydes have been reported [6,7,8,9,10,11,12,13,14,15,16,17,18,19,20,21,22,23,24,25,26,27,28,29,30]. In recent years, the active metals or metal oxides were embedded in various solid supports as a common strategy, for example, polyoxometalates (POMs) [10,11,12], metal-containing mesoporous materials [13,14,15,16,17,18,19,20,21,22,23,24] and metal-containing carbon materials [25,26]. However, these catalysts entail a complicated preparation method and high cost. Alternatively, single-component metal oxides have enough potential to proceed with the oxidation, such as V_2_O_5_ [25], MoO_3_ [27], Fe_2_O_3_, [28,29] and Fe_3_O_4_ [30]. These catalysts were relatively simple to prepare but remained environmentally compatible except for iron oxides.

Titanium dioxide (TiO_2_), one of the most common metal oxides, is used as a cosmetic pigment and as a color additive for food owing to its low cost, nontoxicity, and environmental friendliness [31]. TiO_2_ also demonstrates photocatalytic activity for the degradation of pollutants under water [32] and air [33]. The photocatalytic properties of TiO_2_ originate from the formation of a photogenerated electron and hole, which react with the adsorbed oxygen molecule and water, respectively. The resulting reactive species such as hydroxyl radicals (•OH), superoxide radical anions (•O_2_^−^) and hydroperoxy radicals (•OOH) can oxidize organic molecules. Lachheb et al. reported the photocatalytic oxidation of styrene to produce styrene oxide and benzaldehyde catalyzed by the TiO_2_–H_2_O_2_ system [34]. On the other hand, the combination of TiO_2_ and H_2_O_2_ can generate reactive species for the degradation of organic compounds in the absence of photo-irradiation [35,36]. To the best of our knowledge, however, no study has focused on the use of a non-irradiated TiO_2_–H_2_O_2_ combination for the molecular transformation [34]. Herein, we have developed a method to oxidize olefins over a non-irradiated TiO_2_–H_2_O_2_ combination under cost-effective, nontoxic, and environmentally friendly conditions.

## 2. Results and Discussion

### 2.1. Oxidation of 4-Chlorostyrene with H_2_O_2_ over a TiO_2_ Catalyst

To achieve the oxidation under environmentally friendly conditions, we started screening TiO_2_ catalysts for 4-chlorostyrene **1a** as a model substrate. As is well known, TiO_2_ mainly exists in three types of crystalline phases: rutile, anatase and brookite. To determine which crystalline phase has the best catalytic activity for oxidation, a series of reactions were performed using TiO_2_ catalysts (Table S1). The screening showed that all types of TiO_2_ showed moderate selectivity to obtain corresponding benzaldehyde (**2a**). These results assumed that TiO_2_ has efficient catalytic activity for the oxidation regardless of the type of crystalline phase. In addition, we conducted comparative studies with other oxide catalysts, and as a result, TiO_2_ showed the highest yield for the oxidation of **1a** (Table S2).

Next, we optimized the reaction conditions for the oxidation, as summarized in Table 1. To determine the optimal amount of H_2_O_2_, a series of experiments were performed using 1, 3, 5, and 7 equivalents of H_2_O_2_. The yield of **2a** was improved by increasing of the amount of H_2_O_2_ from 1 to 5 mmol (entries 1–3). Further increasing the amount to 7 mmol reduced the yield (entry 4). The reaction without TiO_2_ showed low conversion and yield (entry 5). To determine the optimal temperature, H_2_O_2_ concentration, solvent, and catalyst loading, a series of experiments were performed under various conditions summarized in Tables S3 and S4, which showed almost the same yield. These results led us to understand that the oxidation was a powerful reaction that various conditions hardly influenced.

Both the conversion and the yield were increased from 1 to 16 h (Figure S1). When the reaction time was extended from 16 to 24 h, the conversion was increased, but the yield decreased because overoxidation of **2a** occurred (entry 6). 4-Chlorobenzoic acid and 4-chlorostyrene oxide were obtained as byproducts at 16 h in 4% and 1% yields, respectively (entry 3). The oxidation of **1a** could be performed in a gram scale to obtain isolated **2a** in 45% yield (entry 7).

### 2.2. Scope and Limitations

In order to evaluate the scope and limitations of the oxidation, the reaction was employed on various olefins under optimal conditions. The results, which are shown in Table 2, reveal several features. It is worth noting that the electronic nature of the substituents at the *ortho*- or *para*-position of styrene influenced the oxidation results. The electron-donating groups inhibited oxidation (entries 3–4), while the electron-withdrawing groups facilitated oxidation to increase the selectivity of **2** (entries 5–6). The influence of the substituted position at the aromatic ring differed little between the *ortho*- and *para*- positions in the yield, indicating that the active species is too small to be affected by steric hindrance (entry 7). The yields decreased when styrenes had the methyl and phenyl groups at the β- and α-positions (entries 8–11). It is considered that the reaction between the active species on TiO_2_ and the β-position of **1** is a key step, as the β-substituent effectively inhibited oxidation. The oxidation of 1-octene **1l** hardly proceeded and the corresponding aldehyde (**2i**) was not detected (entry 12). This result assumed that the oxidation of the olefins that were conjugated with the aromatic rings proceeded to produce the related aldehydes. A series of results indicated that the oxidation was initiated by the generation of benzyl radicals on the catalytic system. The generation of benzyl radicals in the oxidation reaction depended on the electronic properties of the precursor olefins reacted with •OH or •OOH as nucleophilic radicals. Surprisingly, the oxidation of cycloalkenes led to the transformation to the corresponding OOH adducts at the allylic positions instead of causing C=C double bond cleavage (Entries 13–14). These results implied that •OOH/•O_2_^−^ is the one of the reactive species in the oxidation process.

### 2.3. Detection of Active Species for the Oxidation and Catalyst Recyclability

In order to investigate the mechanism underlying the oxidation catalyzed by the non-irradiated H_2_O_2_–TiO_2_ combination, a series of control experiments were performed, as summarized in Table 3. When this reaction was carried out under an argon atmosphere, the yield was almost the same result as under air (entry 2). This clearly indicated that H_2_O_2_, rather than O_2_, worked as the major oxidant for this reaction. To confirm the contribution of TiO_2_ as a photocatalyst, oxidation was performed in complete darkness, resulting in almost the same conversion and yield as entry 1 (entry 3). It was shown that the oxidation did not contribute to the photocatalytic reaction. This was supported by the result of a photocatalytic reaction performed without H_2_O_2_ (entry 4). Although the oxidation proceeded in the presence of light irradiation, the yield was almost the same as entry 1 (entry 5). In the presence of butylhydroxytoluene (BHT) as a well-known radical scavenger, oxidation decreased the conversion from 82% to 11% and the yield from 54% to 1% (entry 6). These results suggested that the cleavage of the C=C double bond is initiated by radical species produced by the reaction with H_2_O_2_ on the TiO_2_ surface. The use of an •OH scavenger *tert*-butyl alcohol (*t*-BuOH) as a solvent instead of acetonitrile (MeCN) showed no effect on oxidation (entry 7). On the other hand, the addition of 1,4-benzoquinone (BQ) as a scavenger for •OOH/•O_2_^–^ led to a decrease in both the conversion and the yield (entry 8) [37]. These results demonstrated that the major reaction pathway for oxidation was through the •OOH/•O_2_^–^ process.

**Figure 1:**
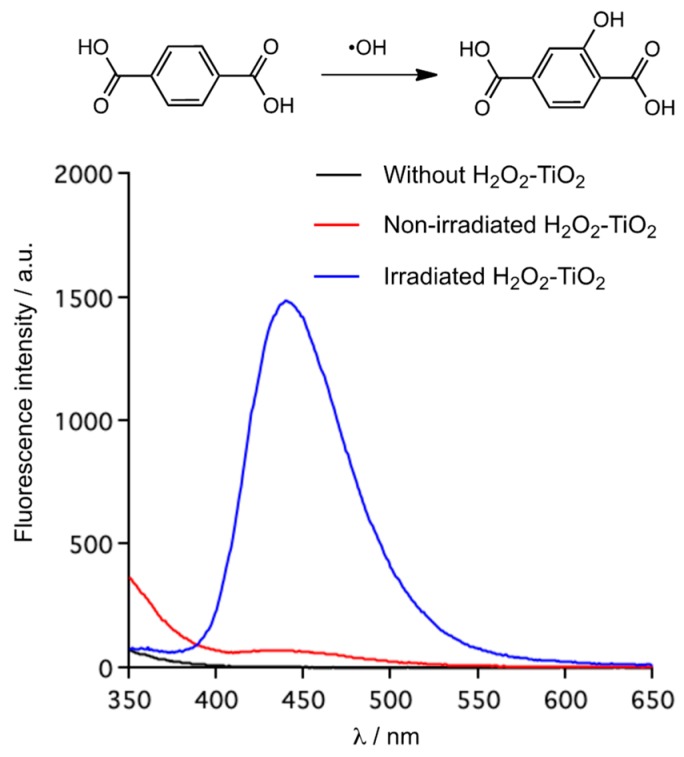
Detection of •OH by the molecular probe method: the formed TAOH fluorescence spectra in MeCN; non-irradiated reaction condition: TA (1 mmol, 170 mg), TiO_2_ P25 (100 mg), 35% H_2_O_2_ (5 equiv.), MeCN (6.7 mL), 80 °C, 1 h; irradiated reaction condition: TA (1 mmol, 170 mg), TiO_2_ P25 (100 mg), 35% H_2_O_2_ (5 equiv.), MeCN (6.7 mL), 80 °C, 1 h, *hv* (365 nm, 10 mW/cm).

To gain further insight into the oxidation, we performed fluorescence probe experiments for detecting •OH. It was reported that the reaction with terephthalic acid (TA) and •OH gave 2-hydroxyterephthalic acid (TAOH), which showed strong fluorescence at 440 nm in MeCN solution [38]. TA acts as just the scavenger for •OH, because of no reaction with other reactive oxygen species such as •OOH, •O_2_^−^ and H_2_O_2_. The fluorescence spectra are shown in Figure 1. On the basis of these spectra, the fluorescence intensity of TAOH formed by the irradiated H_2_O_2_–TiO_2_ system appeared at around 440 nm, indicating that it worked as a photocatalyst to generate •OH. In contrast, the non-irradiated H_2_O_2_–TiO_2_ system showed very weak fluorescence at that region. The two systems differed by an order of magnitude. •OH was probably not a major reactive species in oxidation because it had little influence on the yield of the oxidation of **1a** despite the 20-fold difference in the amount of generated •OH between the irradiated and the non-irradiated H_2_O_2_–TiO_2_ systems. Therefore, it is appropriate that the H_2_O_2_–TiO_2_ combination in dark produced •OOH as the major active species for oxidation. In accord with the literature, the non-irradiated H_2_O_2_–TiO_2_ combination generated •OOH/•O_2_^−^ to degrade methylene blue (MB), and •OOH/•O_2_^−^ was detected by an ESR study using 5,5-dimethyl-1-pyrroline *N*-oxide (DMPO) as a spin trap [35,36]. Three types of reacted H_2_O_2_ with the TiO_2_ surface were identified from the IR spectra [39]. From these studies, we considered that H_2_O_2_ reacted with TiO_2_ to form peroxo-Ti(IV), followed by the generation of •OOH associated with the Ti(IV)/Ti(III) redox process.

**Figure 2:**
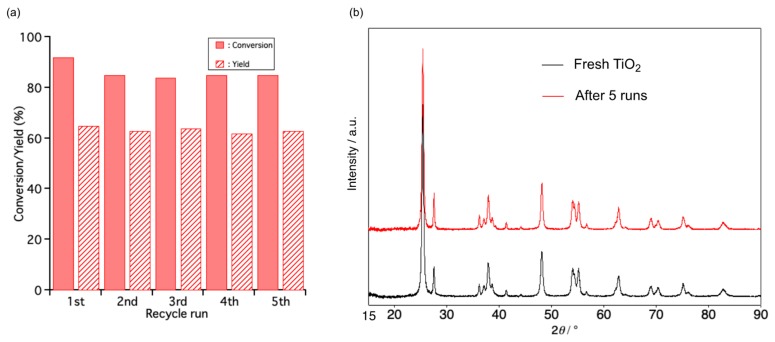
(**a**) The catalytic performance of recycled catalyst for oxidation; Reaction condition: **1a** (1 mmol), TiO_2_ P25 (100 mg), MeCN (7.3 mL). (**b**) XRD patterns of fresh TiO_2_ and five-times-reused TiO_2_.

Recyclability, an important characteristic of heterogeneous catalysts, was evaluated. After each recycle run was performed under optimal conditions, the catalyst was recovered by centrifugation and washed with MeCN, then dried at 110 °C overnight. The catalytic results of TiO_2_ after each recycle run showed little difference compared with a fresh one (Figure 2a). No change in the XRD patterns between five-times-reused and fresh catalyst showed that using the catalytic system preserved the catalyst’s structure (Figure 2b). This result demonstrated that the catalytic system for oxidation had significant stability and recyclability. When the catalyst was removed from the reaction solution at 50 °C after 1 h, no further reaction occurred (Figure S2). This result suggested that the reaction occurred on the surface of the TiO_2_ catalyst.

## 3. Conclusions

In conclusion, we have developed a method for the TiO_2_-catalyzed thermal oxidation of styrenes to the corresponding benzaldehydes using H_2_O_2_ as an oxidant in good yield. Our oxidation method using the combination of green catalyst TiO_2_ and green oxidant H_2_O_2_ made UV irradiation unnecessary and allowed us to efficiently cleave the C=C double bond of styrenes along with the generation of radical species. Notably, unlike a photocatalytic reaction, oxidation efficiently proceeded regardless of the type of crystalline phase of TiO_2_. From the fluorescence probe and the competitive scavenging experiments, •OOH/•O_2_^−^ are thought to be key active species for oxidation derived from the thermal H_2_O_2_ reaction with TiO_2_. In addition, our oxidation protocol allowed the reuse of the catalyst and ease of purification. Therefore, these conditions provided cost effectiveness, non-toxicity, and environmental compatibility. Our method should be highly feasible for industrial applications. Investigation of the detail of the reaction mechanism is under way.

## Figures and Tables

**Table 1:**
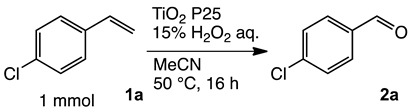
Optimization of the reaction conditions.

Entry	TiO_2_ (mg)	H_2_O_2_ (mmol)	Conversion (%) ^a^	Yield (%) ^a^
1	100	1	47	31
2	100	3	86	60
3	100	5	92	65
4	100	7	90	56
5	0	5	8	1
6 ^b^	100	5	98	63
7 ^c^	100	5	85	45 ^d^

^a^ All conversions and yields were determined on the basis of **1a** by GC-FID using biphenyl as an internal standard. ^b^ For 24 h. ^c^ Reaction conditions: **1a** (7.3 mmol, 1 g), TiO_2_ (730 mg), MeCN (7.3 mL). ^d^ Isolated yield.

**Table 2:**
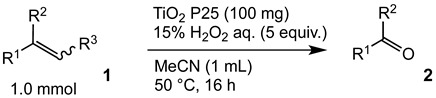
The oxidation of various olefins with H_2_O_2_ over a TiO_2_ catalyst.

Entry	Substrate	Product	Conversion (%) ^a^	Yield (%) ^a^
1	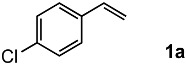	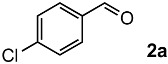	92	65
2	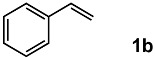	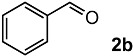	82	54
3	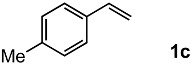	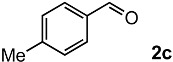	92	57
4	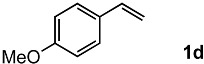	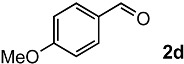	63	23
5	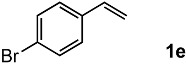	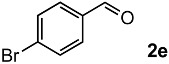	91	65
6	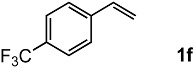	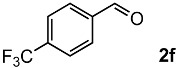	79	58
7	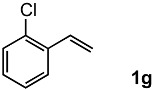	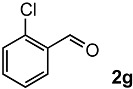	92	72
8	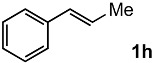	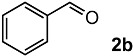	35	15
9	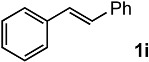	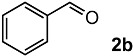	22	7
10	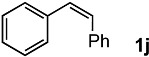	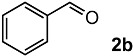	5	3
11	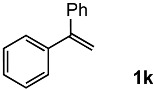	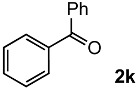	76	43
12	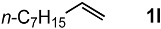	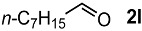	7	0
13	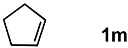	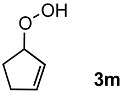	84	30 ^b^
14	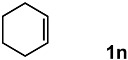	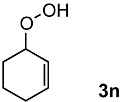	65	23 ^b^

^a^ Conversions and yields were determined on the basis of **1** by GC-FID using biphenyl as an internal standard. ^b^ Yields were determined by NMR spectra using biphenyl as an internal standard.

**Table 3:**
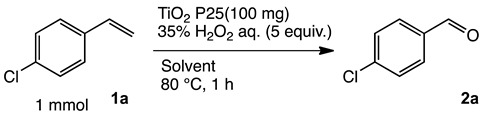
Control experiments for the oxidation.

Entry	Additive	Atmosphere	Irradiation	Solvent	Conversion (%) ^a^	Yield (%) ^a^
1	-	Air	Room light	MeCN	82	31
2	-	Argon	Room light	MeCN	82	51
3	-	Air	Darkness	MeCN	83	52
4 ^b^	-	Air	*hv *(365 nm) ^c^	MeCN	1	0
5	-	Air	*hv* (365 nm) ^c^	MeCN	91	51
6	BHT (1 equiv.)	Air	Room light	MeCN	11	1
7	-	Air	Room light	*t*-BuOH	85	55
8	BQ (0.5 equiv.)	Air	Room light	MeCN	2	1

^a^ All conversions and yields were determined on the basis of **1a** by GC-FID using biphenyl as an internal standard. ^b^ Addition of water instead of H_2_O_2_. ^c^ Irradiated intensity: 10 mW/cm^2^.

## Supplementary Materials

The following are available online, Table S1: Comparison of the type of TiO2 catalysts for the oxidation; Table S2: Comparison of catalysts for the oxidation; Table S3: Additional optimization of the reaction conditions; Table S4: Comparison of various solvents for the oxidation; Figure S1: Time profile of the oxidation; reaction condition: **1a** (1 mmol), TiO_2_ (100 mg), 15% H_2_O_2_ (5 equiv.), MeCN (7.3 mL), 50 °C; Figure S2: The hot filtration experiment for the oxidation with TiO_2_ catalyst; reaction condition: **1a** (1 mmol), TiO_2_ (100 mg), 15% H_2_O_2_ (5 equiv.), MeCN (7.3 mL), 50 °C.

## References

[1] Bäckvall J.-E. (2004). Modern Oxidation Methods.

[2] Diaper D.G. (1955). Ozonolysis of 1-substituted cycloolefins. Can. J. Chem..

[3] Pappo R., Allen D.S., Lemieux R.U., Johnson W.S. (1956). Osmium Tetroxide-Catalyzed Periodate Oxidation of Olefinic Bonds. J. Org. Chem..

[4] Spannring P., Bruijnincx P.C.A., Weckhuysen B.M., Gebbink R.J.M. (2014). Transition metal-catalyzed oxidative double bond cleavage of simple and bio-derived alkenes and unsaturated fatty acids. Catal. Sci. Technol..

[5] Urgoitia G., SanMartin R., Herrero M.T., Domínguez E. (2017). Aerobic Cleavage of Alkenes and Alkynes into Carbonyl and Carboxyl Compounds. ACS Catal..

[6] Reddy J.S., Khire U.R., Ratnasamy P., Mitra R.B. (1992). Cleavage of the Carbon-Carbon Bond over Zeolites using Hydrogen Peroxide. J. Chem. Soc. Chem. Commun..

[7] Tong J., Li W., Bo L., Wang H., Hu Y., Zhang Z., Mahboob A. (2016). Selective oxidation of styrene catalyzed by cerium-doped cobalt ferrite nanocrystals with greatly enhanced catalytic performance. J. Catal..

[8] Adam F., Iqbal A. (2011). The liquid phase oxidation of styrene with tungsten modified silica as a catalyst. Chem. Eng. J..

[9] Ghosh S., Acharyya S.S., Bal R. (2015). One-pot preparation of nanocrystalline Ag–WO_3_ catalyst for the selective oxidation of styrene. RSC Adv..

[10] Sun W., Hu J. (2016). Oxidation of styrene to benzaldehyde with hydrogen peroxide in the presence of catalysts obtained by the immobilization of H_3_PW_12_O_40_ on SBA-15 mesoporous material. Reac. Kinet. Mech. Cat..

[11] Pathan S., Patel A. (2013). Transition-Metal-Substituted Phosphomolybdates: Catalytic and Kinetic Study for Liquid-Phase Oxidation of Styrene. Ind. Eng. Chem. Res..

[12] Sharma P., Patel A. (2009). Chemical Supported 12-molybdophosphoricacid: Characterization and non-solvent liquid phase oxidation of styrene. J. Mol. Catal. A Chem..

[13] Zhang L., Hua Z., Dong X., Li L., Chen H., Shi J. (2007). Preparation of highly ordered Fe-SBA-15 by physical-vapor-infiltration and their application to liquid phase selective oxidation of styrene. J. Mol. Catal. A Chem..

[14] Hulea V., Dumitriu E. (2004). Styrene oxidation with H_2_O_2_ over Ti-containing molecular sieves with MFI, BEA and MCM-41 topologies. Appl. Catal. A.

[15] Wang Y., Zhang Q., Shishido T., Takehira K. (2002). Characterizations of Iron-Containing MCM-41 and Its Catalytic Properties in Epoxidation of Styrene with Hydrogen Peroxide. J. Catal..

[16] Yang Y., Zhang Y., Hao S., Guan J., Ding H., Shang F. (2010). Heterogenization of functionalized Cu (II) and VO (IV) Schiff base complexes by direct immobilization onto amino-modified SBA-15: Styrene oxidation catalysts with enhanced reactivity. Appl. Catal. A.

[17] Li B., Zhu Y., Jin X. (2015). Synthesis of cobalt-containing mesoporous catalysts using the ultrasonic-assisted “pH-adjusting” method: Importance of cobalt species in styrene oxidation. J. Solid State Chem..

[18] Wang H., Qian W., Chen J., Wu Y., Xu X., Wang J., Kong Y. (2014). Spherical V-MCM-48: The synthesis, characterization and catalytic performance in styrene oxidation. RSC Adv..

[19] Chen D., Li N., Sun P., Kong Y. (2009). Catalytic Performance of Ti-MCM-41 for Styrene Oxidation. Chin. J. Catal..

[20] Zhang Y., Shen J., Zhu J., Sun Y. (2012). Study on preparation process of benzaldehyde by catalytic oxidation of styrene on catalyst V-SBA-15. Petrochem. Tech. Appl..

[21] Gao D., Gao Q. (2007). Selective oxidation of styrene to benzaldehyde over VSB-5 and isomorphously substituted cobalt VSB-5. Catal. Commun..

[22] Maurya M.R., Chandrakar A.K., Chand S. (2007). Oxidation of phenol, styrene and methyl phenyl sulfide with H_2_O_2_ catalysed by dioxovanadium (V) and copper (II) complexes of 2-aminomethylbenzimidazole-based ligand encapsulated in zeolite-Y. J. Mol. Catal. A Chem..

[23] Tanglumlert W., Imae T., White T.J., Wongkasemjit S. (2009). Styrene oxidation with H_2_O_2_ over Fe- and Ti-SBA-1 mesoporous silica. Catal. Commun..

[24] Campelo J.M., Conesa D., Gracia J., Jurado J., Luque R., Maria J., Angel A. (2008). Microwave facile preparation of highly active and dispersed SBA-12 supported metal nanoparticles. Green Chem..

[25] Zou H., Hu C., Chen K., Xiao G., Peng X. (2018). Cobalt vanadium oxide supported on reduced graphene oxide for the oxidation of styrene derivatives to aldehydes with hydrogen peroxide as oxidant. Synlett.

[26] Zou H., Xiao G., Chen K., Peng X. (2018). Noble metal-free V_2_O_5_/g-C_3_N_4_ composites for selective oxidation of olefins using hydrogen peroxide as an oxidant. Dalton Trans..

[27] Jafarpour M., Ghahramaninezhad M., Rezaeifard A. (2014). Catalytic activity and selectivity of reusable α- MoO_3_ nanobelts toward oxidation of olefins and sulfides using economical peroxides. RSC Adv..

[28] Shi F., Tse M.K., Pohl M., Brückner A., Zhang S., Beller M. (2007). Tuning Catalytic Activity between Homogeneous and Heterogeneous Catalysis: Improved Activity and Selectivity of Free Nano-Fe_2_O_3_ in Selective Oxidations. Angew. Chem. Int. Ed..

[29] Shi F., Kin M., Pohl M., Radnik J., Brückner A., Zhang S., Beller M. (2008). Nano-iron oxide-catalyzed selective oxidations of alcohols and olefins with hydrogen peroxide. J. Mol. Catal. A Chem..

[30] Xie L., Wang H., Lu B., Zhao J. (2018). Highly selective oxidation of styrene to benzaldehyde over Fe_3_O_4_ using H_2_O_2_ aqueous solution as oxidant. Reac. Kinet. Mech. Cat..

[31] Carp O., Huisman C.L., Reller A. (2004). Photoinduced reactivity of titanium dioxide. Prog. Solid State Chem..

[32] Mills A., Davies R.H., Worsley D. (1993). Water Purification by Semiconductor Photocatalysis. Chem. Soc. Rev..

[33] Ren H., Koshy P., Chen W., Qi S., Sorrell C.C. (2017). Photocatalytic materials and technologies for air purification. J. Hazard. Mater..

[34] Lachheb H., Guillard C., Lassoued H., Haddaji M., Rajah M., Houas A. (2017). Photochemical oxidation of styrene in acetonitrile solution in presence of H_2_O_2_, TiO_2_/H_2_O_2_ and ZnO/H_2_O_2_. J. Photochem. Photobiol. A.

[35] Wiedmer D., Sagstuen E., Welch K., Haugen H.J., Tiainen H. (2016). Oxidative power of aqueous non-irradiated TiO_2_-H_2_O_2_ suspensions: Methylene blue degradation and the role of reactive oxygen species. Appl. Catal. B.

[36] Sánchez L.D., Taxt-lamolle S.F.M., Hole E.O., Krivokapić A., Sagstuen E., Haugen H.J. (2013). TiO_2_ suspension exposed to H_2_O_2_ in ambient light or darkness: Degradation of methylene blue and EPR evidence for radical oxygen species. Appl. Catal. B.

[37] Gong Y., Wang D.P., Wu R., Gazi S., Soo H.S., Sritharan T., Chen Z. (2017). New insights into the photocatalytic activity of 3-D core–shell P25@silica nanocomposites: Impact of mesoporous coating. Dalton Trans..

[38] Wang W., Hu S., Li L., Gao W., Cong R., Yang T. (2017). Octahedral-based redox molecular sieve M-PKU-1: Isomorphous metal-substitution, catalytic oxidation of *sec*-alcohol and related catalytic mechanism. J. Catal..

[39] Nosaka Y., Nosaka A.Y. (2017). Generation and Detection of Reactive Oxygen Species in Photocatalysis. Chem. Rev..

