# Supplementary material for: Titania-Catalyzed H2O2 Thermal Oxidation of Styrenes to Aldehydes
Source: Molecules. 2019 Jul 10;24(14):2520. doi: 10.3390/molecules24142520 (PMC6680574; doi:10.3390/molecules24142520)

Supporting Information

Titania-Catalyzed H_2O_2 Thermal Oxidation of Styrenes to Aldehydes

Satoru Ito [1,*], Yoshihiro Kon [2,*], Takuya Nakashima [2], Dachao Hong [2], Hideo Konno [2], Daisuke Ino [1] and Kazuhiko Sato [2,*]

[1] Institute for Energy and Material/Food Resources, Technology Innovation Division, Panasonic Corporation, 3-4 Hikaridai, Seika-cho, Soraku-gun, Kyoto 619-0237, Japan; ino.daisuke@jp.panasonic.com (D.I.)

[2] Interdisciplinary Research Center for Catalytic Chemistry, National Institute of Advanced Industrial Science and Technology (AIST), Central 5, 1-1-1 Higashi, Tsukuba, Ibaraki 305-8565, Japan; takuya-nakashima@aist.go.jp (T.N.); hong-d@aist.go.jp (D.H.); h-konno@aist.go.jp (H.K.)

* Correspondence: ito.satoru002@jp.panasonic.com (S.I.); y-kon@aist.go.jp (Y.K.); k.sato@aist.go.jp (K.S.)

Received: 1 June 2019; Accepted: 9 July 2019; Published: date
Academic Editor: Kei Saito

Instrumentation and Materials

Gas chromatography (GC) analyses were performed on a Shimadzu GC-2014 using a Inert-Cap-1 column (0.25 mm x 30 m, GL Sciences Inc.). ^{1}H NMR (400 MHz) and ^{13}C NMR (100 MHz) spectra were recorded on a JEOL ECX-400P spectrometer at 298 K. Chemical shifts (δ) are in parts per million relative to tetramethylsilane (TMS) at 0.00 ppm for ^{1}H and relative to residual $CHCl_3$ at 77.0 ppm for ^{13}C unless otherwise noted. X-ray diffraction (XRD) patterns were collected using a Rigaku MiniFlex 600 diffractometer equipped with a Cu-Kα radiation. All powder samples were scanned over 2θ range 15° to 90° with the ratio of 0.01° / sec. Fluorescence spectra were recorded on a Hamamatsu photonics C9920-02 absolute PL quantum yield measurement system.

All the reagents for synthesis and characterization analysis were used without further purification. Titanium(IV) oxide (TiO_2) with a crystalline phase anatase (82%) and rutile (15%) commercialized under the name P25 was obtained from Evonik Industries[S1]. JRC-TIO-1 (anatase-TiO_2), rutile-TiO_2 (JRC-TIO-16), silicon(IV) oxide (SiO_2: JRC-SIO-1), aluminium(III) oxide (γ-Al_2O_3: JRC-ALO-6) and Cerium(IV) oxide (CeO_2: JRC-CEO-5) were kindly supplied from the Catalysis Society of Japan (CSJ). The commercial brookite-TiO_2 (TIO19PB) and tantalum(V) oxide (Ta_2O_5: TA002PB) were purchased from Kojundo Chemicals, Ltd. Zirconium(IV) oxide (ZrO_2: RC-100) was obtained from Daiichi Kigenso Kagaku Kogyo Co., Ltd. 4-Chlorostyrene, styrene, 4-methoxystyrene, 4-methylstyrene, 4-bromostyrene, 4-(trifluoromethyl)styrene, 1,1-diphenylethylene, 1-nonene, cyclopentene, cyclohexene, and 1,4-benzoquinone were obtained from Tokyo Chemical Industry Co., Ltd. 4-chloro-*α*-methylstyrene, 2-chlorostyrene, terephthalic acid, dibutylhydroxytoluene, biphenyl, acetonitrile, ethanol, ethyl acetate, 1,4-dioxane, dimethylformamide, benzonitrile, toluene, *t*-butyl alcohol and $CDCl_3$ were obtained from FUJIFILM Wako Pure Chemical Corporation. Iron(III) oxide (Fe_2O_3: 310050), *trans*-*β*-methylstyrene were obtained from Sigma-Aldrich. Hydrogen peroxide was obtained from Kanto Chemical Co., Inc.

General Procedures and Compound Data

Typical procedures for H_2O_2 thermal oxidation.

The oxidation of styrenes was performed in a 15 mL test tube. 0.10 g (1 mmol) styrene was stirred with 100 mg TiO_2 in 1.0 mL acetonitrile. Subsequently, 1.0 ml H_2O_2 (15 wt%) was added into the mixture. The resulting mixture was stirred at 50 ºC for 16 h. After reaction, the solution was cooled to room temperature, then diluted with 12 mL of

acetonitrile and filtered. Biphenyl (40 mg, 0.25 mmol) was added to the solution as an internal standard for gas chromatography (GC) analysis. The solution was placed under ultrasonic irradiation for 10 min to ensure a good homogeneity of the mixture. The conversion and yield were determined on the basis of the analysis of the mixture by GC.

Oxidation of 2-chlorostyrene to 2-chlorobenzaldehyde in 1g scale.

The oxidation of 2-chlorostyrene was performed in a 50 mL test tube. 1.0 g (7.3 mmol) 2-chlorostyrene was stirred with 730 mg TiO_2 in 7.3 mL acetonitrile. Subsequently, 7.3 ml H_2O_2 (15 wt%) was added into the mixture. The resulting mixture was stirred at 50 °C for 16 h. The catalyst was separated by centrifugation and product was extracted with toluene and saturated sodium chloride solution. The organic phase was dried with anhydrous sodium sulfate and purified by distillation under reduced pressure (98-100 °C at 13 torr), to give 0.46 g of 2-chlorobenzaldehyde in 45% yield (81% conversion).

Oxidation of terephthalic acid to 2-hydroxyterephthalic acid.

The oxidation of terephthalic acid was performed in a 15 mL test tube. 0.17 g (1 mmol) terephthalic acid was stirred with 100 mg TiO_2 in 6.7 mL acetonitrile. Subsequently, 0.44 ml H_2O_2 (35 wt%) was added into the mixture. The resulting mixture was stirred at 80 °C for 1 h. After reaction, the solution was cooled to room temperature and filtered.

Compound data of products

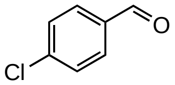

2a

4-Chlorobenzaldehyde 2a[S2]: ^{1}H NMR (400 MHz, $CDCl_3$): δ 9.99 (s, 1H), 7.83 (d, *J* = 8.0 Hz, 2H), 7.52 (d, *J* = 8.8 Hz, 2H); ^{13}C NMR (100 MHz, $CDCl_3$): δ 190.9, 141.0, 134.8, 131.0, 129.5.

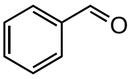

2b

Benzaldehyde 2b[S2]: ^{1}H NMR (400 MHz, $CDCl_3$): δ 10.02 (s, 1H), 7.90-7.87 (m, 2H), 7.65-7.61 (m, 1H), 7.53 (t, *J* = 7.6 Hz, 2H); ^{13}C NMR (100 MHz, $CDCl_3$): δ 192.4, 136.5, 134.5, 129.8, 129.1.

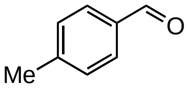

2c

***p*-Tolualdehyde 2c**[S2]: ^{1}H NMR (400 MHz, $CDCl_3$): δ 9.96 (s, 1H), 7.77 (d, *J* = 7.6 Hz,

2H), 7.32 (d, *J* = 8.0 Hz, 2H), 2.43 (s, 3H); ^{13}C NMR (100 MHz, $CDCl_3$): δ 192.0, 145.6, 134.3, 129.9, 129.8, 21.9.

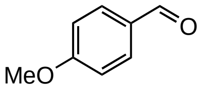

2d

***p*-Anisaldehyde 2d**[S2]: ^{1}H NMR (400 MHz, $CDCl_3$): δ 9.89 (s, 1H), 7.85 (d, *J* = 8.8 Hz, 2 H), 7.01 (d, *J* = 8.8 Hz, 2H), 3.90 (s, 3H); ^{13}C NMR (100 MHz, $CDCl_3$): δ 190.9, 164.7, 132.0, 130.0, 114.4, 55.6.

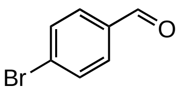

2e

4-Bromobenzaldehyde 2e[S2]: ^{1}H NMR (400 MHz, $CDCl_3$): δ 9.99 (s, 1H), 7.77-7.75 (m, 2H), 7.71-7.69 (m, 2H); ^{13}C NMR (100 MHz, $CDCl_3$): δ 191.1, 135.2, 132.5, 131.1, 129.9.

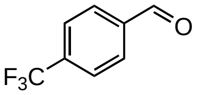

2f

4-Trifluoromethylbenzaldehyde 2f[S3]: ^{1}H NMR (400 MHz, $CDCl_3$): δ 10.11 (s, 1H), 8.01 (d, *J* = 8.4 Hz, 2H), 7.81 (d, *J* = 8.4 Hz, 2H); ^{13}C NMR (100 MHz, $CDCl_3$): δ 191.3, 138.9, 135.7 (q, J = 32.4 Hz), 130.1, 126.2(q, J = 3.8 Hz), 123.5 (q, J = 271.8 Hz).

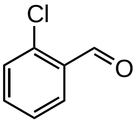

2g

2-Chlorobenzaldehyde 2g[S4]: ^{1}H NMR (400 MHz, $CDCl_3$): δ 10.49 (s, 1H), 7.93 (dd, *J* = 8.0 Hz, 1.6 Hz, 1H), 7.55-7.37 (m, 3H); ^{13}C NMR (100 MHz, $CDCl_3$): δ 189.8, 138.0, 135.2, 132.6, 130.7, 129.4, 127.4.

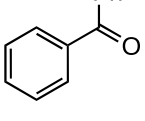

2k

Benzophenone 2k[S5]: ^{1}H NMR (400 MHz, $CDCl_3$): δ 7.82-7.80 (m, 8H), 7.59 (t, *J* = 7.6 Hz, 4H), 7.49 (t, *J* = 7.6 Hz, 8H); ^{13}C NMR (100 MHz, $CDCl_3$): δ 196.8, 137.7, 132.5, 130.1, 128.4.

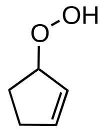

3m

3-Hydroperxycyclopent-1-ene 3m[S6]: ^{1}H NMR (400 MHz, $CDCl_3$): δ 8.06 (br, 1H), 6.17 (m, 1H), 5.83 (m, 1H), 5.16 (m, 1H), 2.51-1.90 (m, 4H); ^{13}C NMR (100 MHz, $CDCl_3$): δ 139.2, 128.0, 90.9, 31.2, 27.9.

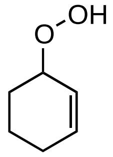

3n

3-Hydroperxycyclohex-1-ene 3n[S7]: ^{1}H NMR (400 MHz, $CDCl_3$): δ 8.21 (br, 1H), 6.04 (m, 1H), 5.79 (m, 1H), 4.52 (m, 1H), 2.11-1.91 (m, 3H), 1.80-1.55 (m, 3H); ^{13}C NMR (100 MHz, $CDCl_3$): δ 134.3, 124.0, 78.4, 26.3, 25.3, 18.4.

Table S1. Comparison of the type of TiO_2 catalysts for the oxidation.

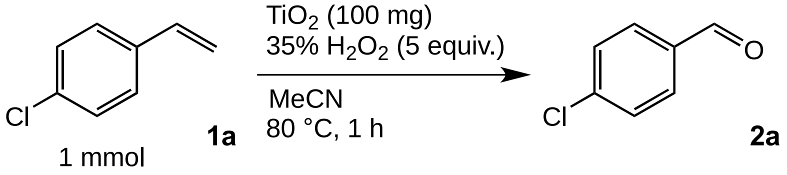

Entry	TiO_2	Crystalline phase	SSA (m^2/g)[a]	Conversion (%)[b]	Selectivity (%)[c]	Yield (%)[b]
1	P-25	Anatase/rutile	63[S1]	82	66	54
2	JRC-TIO-1	Anatase	73[S1]	63	63	49
3	JRC-TIO-16	Rutile	110[S8]	74	64	47
4	TIO19PB	Brookite	23[S9]	80	59	47

[a] Specific surface area (BET). [b] All conversions and yields were determined on the basis of **1a** by GC-FID using biphenyl as an internal standard. [c] All selectivities for **2a** were calculated by the following formula: selectivity = yield / conversion × 100.

Table S2. Comparison of catalysts for the oxidation.

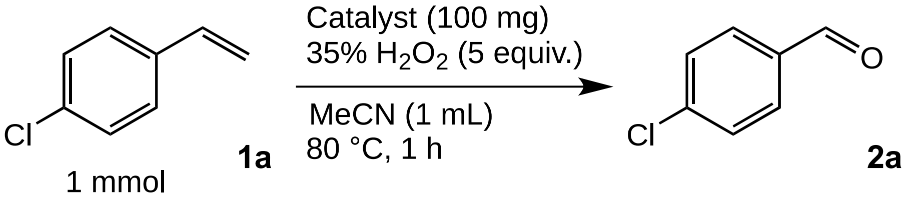

Entry	Catalyst	Conversion (%)[a]	Yield (%)[a]
1	TiO_2 P25	82	54
2	ZrO_2	3	2
3	Ta_2O_5	4	4
4	g-Al_2O_3	11	2
5	SiO_2	13	1
6	CeO_2	0	0
7	Fe_2O_3	7	4

[a] All conversions and yields were determined on the basis of **1a** by GC-FID using biphenyl as an internal standard.

Table S3. Additional optimization of the reaction conditions.

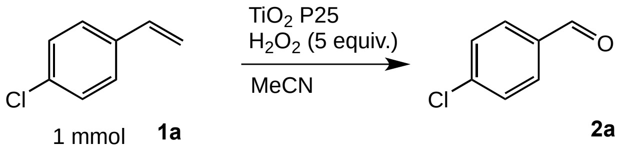

Entry	TiO_2 (mg)	H_2O_2 aq. Concentration (%)	Temperature (ºC)	Time (h)	Conversion (%)[a]	Selectivity (%)[b]	Yield (%)[a]
1	100	35	80	1	82	66	54
2	100	35	80	2	90	60	54
3	100	35	50	16	93	66	61
4	100	35	30	16	61	72	44
5	100	15	50	16	92	71	65
6	100	10	50	16	84	64	54
7	20	15	50	16	76	70	53
8	150	15	50	16	97	65	63

[a] All conversions and yields were determined on the basis of **1a** by GC-FID using biphenyl as an internal standard. [b] All selectivities for **2a** were calculated by the following formula: selectivity = yield / conversion × 100.

Table S4. Comparison of various solvents for the oxidation.

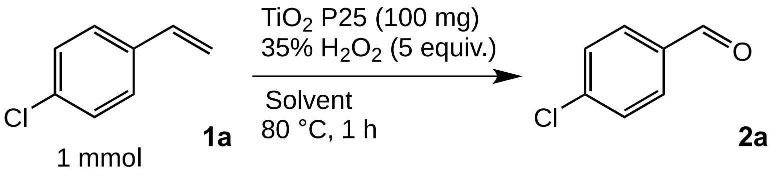

Entry	Solvent	Conversion (%)[a]	Selectivity (%)[b]	Yield (%)[a]
1	MeCN	82	66	54
2	EtOH	89	58	52
3	*t*-BuOH	85	65	55
4	EtOAc	1	-	1
5	1,4-Dioxane	95	57	54
6	DMF	66	42	28
7	PhCN	1	-	1

[a] All conversions and yields were determined on the basis of **1a** by GC-FID using biphenyl as an internal standard. [b] All selectivities for **2a** were calculated by the following formula: selectivity = yield / conversion × 100.

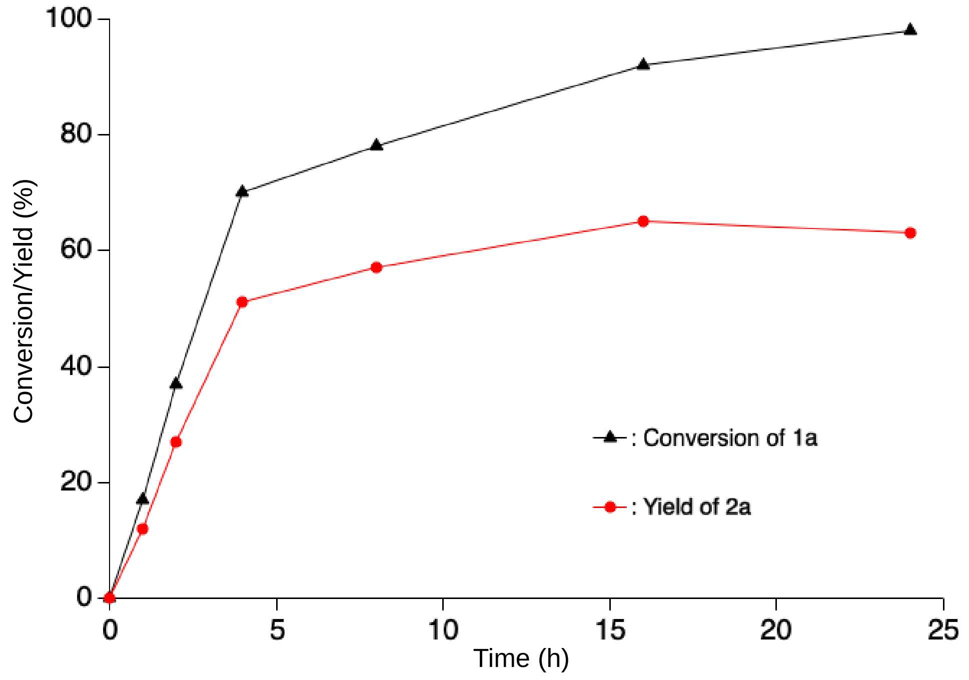

Figure S1. Time profile of the oxidation; reaction condition: **1a** (1 mmol), TiO_2 (100 mg), 15% H_2O_2 (5 equiv.), MeCN (7.3 mL), 50 °C.

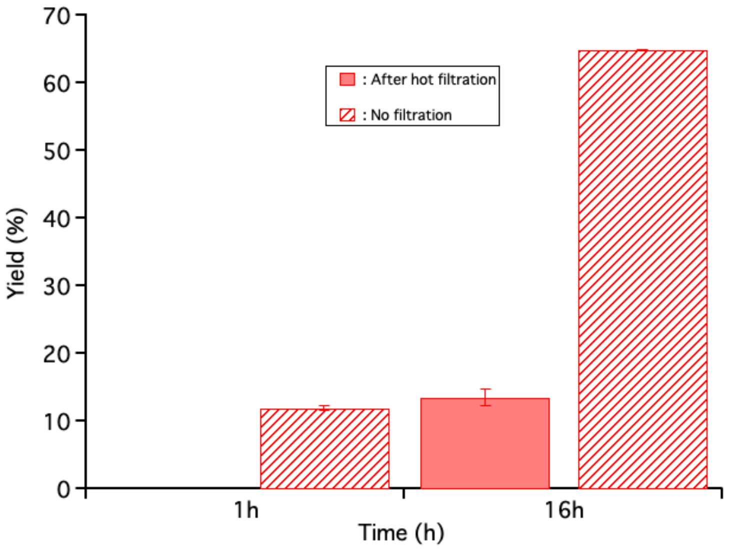

Figure S2. The hot filtration experiment for the oxidation with TiO_2 catalyst; reaction condition: **1a** (1 mmol), TiO_2 (100 mg), 15% H_2O_2 (5 equiv.), MeCN (7.3 mL), 50 °C.

[S1] A. Nitta, M. Takashima, M. Takase, B. Ohtani *Catal. Today* **2019**, *321*, 2-8.
[S2] J. Zheng, S. Lin, X. Zhu, B. Jiang, Z. Yang, Z. Pan *Chem. Commun.* **2012**, *48*, 6235–6237.
[S3] S. Gaspa, A. Porcheddu, A. Valentoni, S. Garroni, S. Enzo, L. D. Luca *Eur. J. Org. Chem.* **2017**, *37*, 5519-5526.
[S4] Q. Feng, Q. Song *J. Org. Chem.* **2014**, *79*, 1867-1871.
[S5] S. Seo, J. B. Taylor, M. F. Greaney *Chem. Commun*. **2012**, *48*, 8270–8272.
[S6] H. Goksu, D. Dalmizrak, S. Akbayrak, M. S. Gultekin, S. Ozkar, O. Metin, *J. Mol. Catal. A: Chem.* **2013**, *378*, 142-147.
[S7] T. Kuga, Y. Sasano, Y. Iwabuchi *Chem. Commun*. **2018**, *54,* 798-801.
[S8] Japan Reference Catalyst. Available online: http://www.shokubai.org/com/sansyo/titania.html (accessed on 29 May 2019).
[S9] J. J. M. Vequizo, H. Matsunaga, T. Ishiku, S. Kamimura, T. Ohno, A Yamakata *ACS catal.* **2017**, *7*, 2644-2651.

^{1}H and ^{13}C NMR Spectra

3-Hydroperxycyclopent-1-ene **3m**

^{1}H NMR (400 MHz, $CDCl_3$, 25 °C)

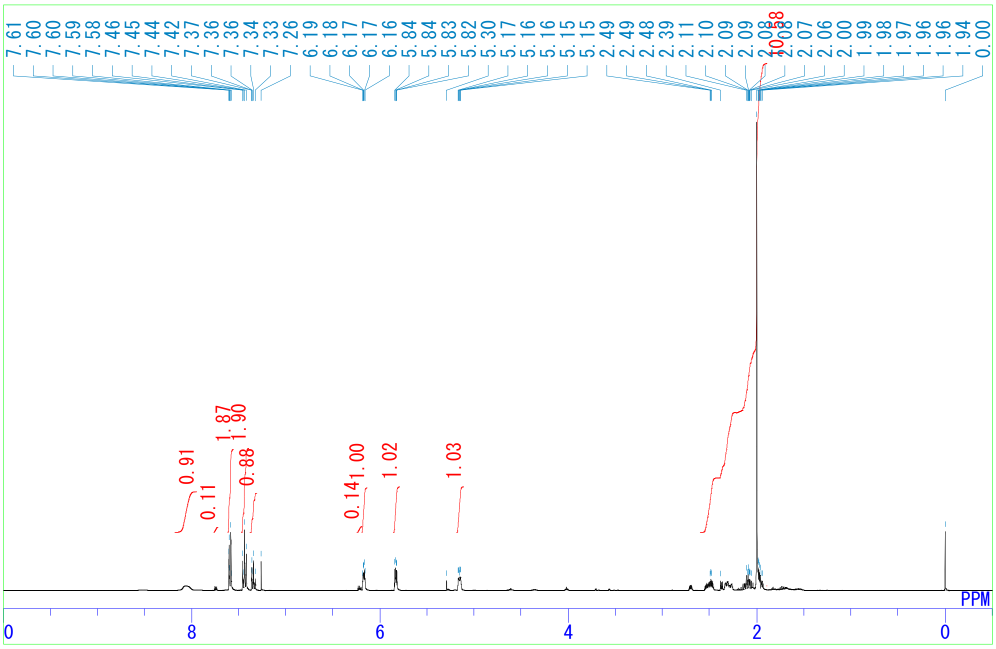

^{13}C NMR (100 MHz, $CDCl_3$, 25 °C)

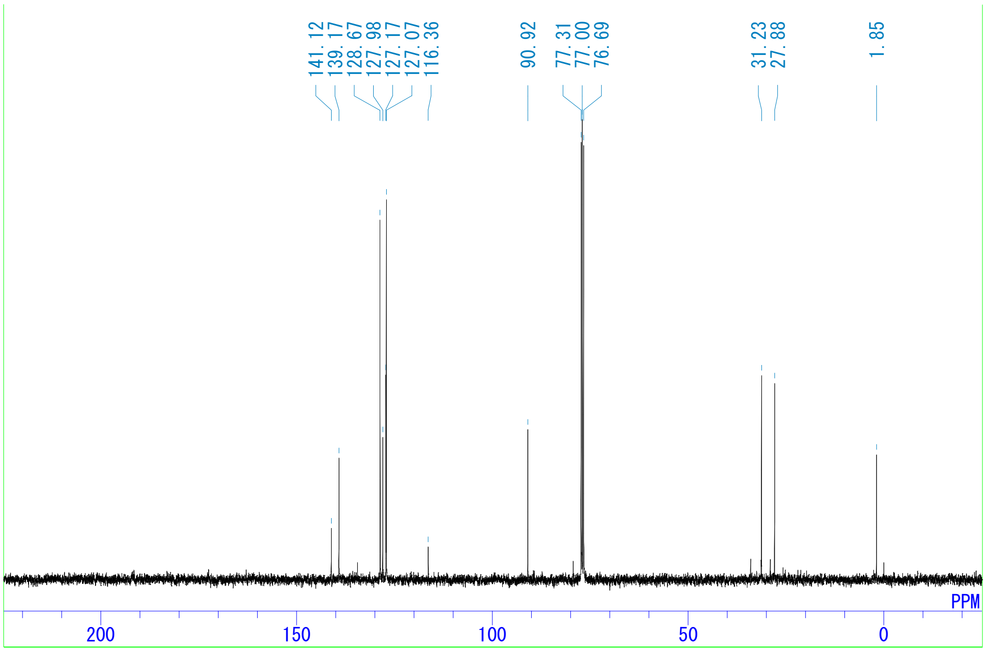

3-Hydroperxycyclohex-1-ene **3n**

^{1}H NMR (400 MHz, $CDCl_3$, 25 °C)

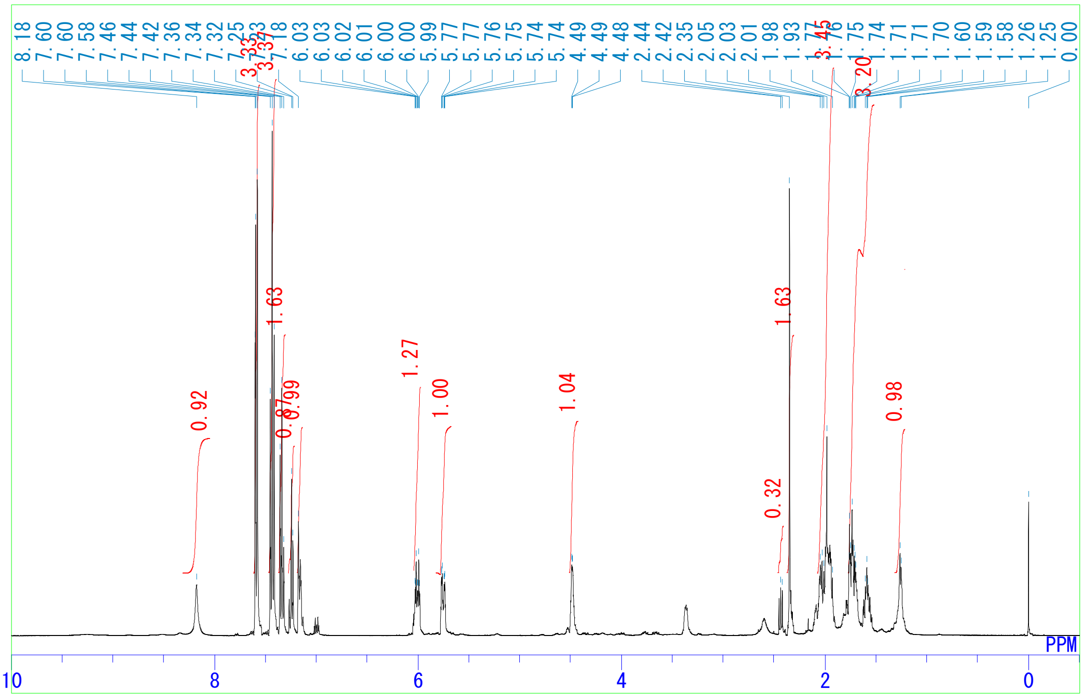

^{13}C NMR (100 MHz, $CDCl_3$, 25 °C)

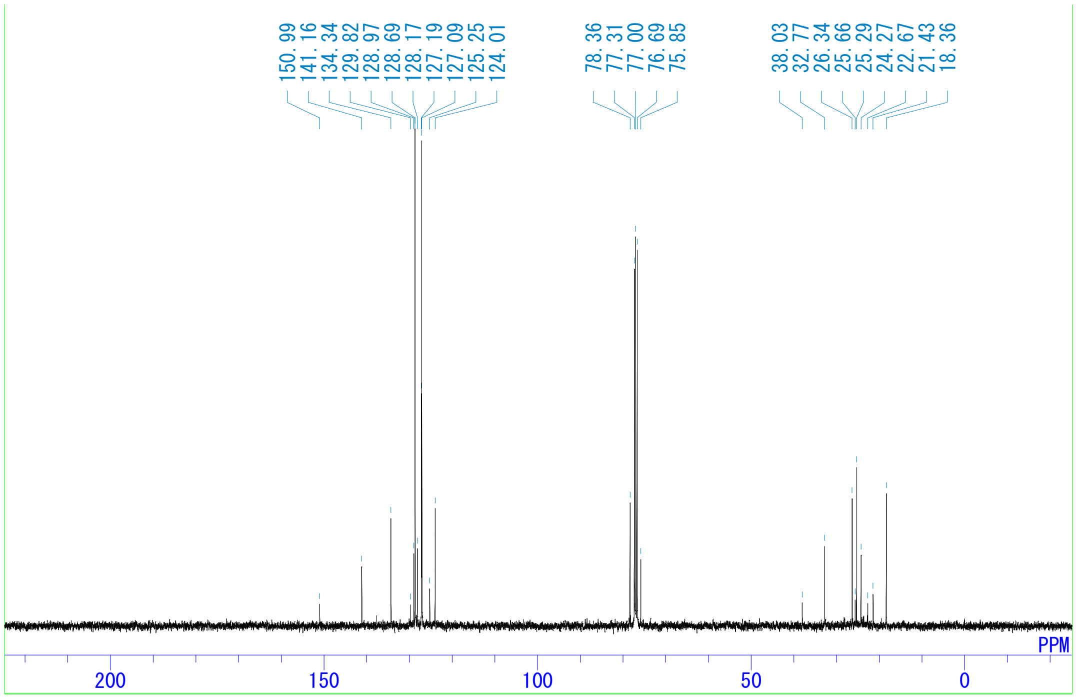

Supplement: Supplementary file 1 [file molecules-24-02520-s001.pdf]
